# Social-networks use as adaptive or maladaptive strategy for coping with stress

**DOI:** 10.1038/s41598-023-39042-4

**Published:** 2023-07-23

**Authors:** Elisa Wegmann, Johannes Schiebener, Matthias Brand

**Affiliations:** 1 Department of General Psychology: Cognition and Center for Behavioral Addiction Research (CeBAR), University of Duisburg-Essen, Forsthausweg 2, 47057 Duisburg, Germany; 2 Erwin L. Hahn Institute for Magnetic Resonance Imaging, Essen, Germany

**Keywords:** Human behaviour, Risk factors

## Abstract

Social networks are frequently used to distract, procrastinate, or cope with stress. We aimed to investigate how (problematic) social-networks use affect stress perception in interaction with different stress recovery conditions. A total of 104 participants were randomly assigned to one of four groups. Three groups underwent a stress induction with subsequent stress recovery via (1) using Facebook, (2) reading magazines, or (3) waiting. Another group (4) waited without stress induction. Stress perception was repeatedly assessed with the State-Trait-Anxiety-Inventory. Facebook use and reading magazines decreased acute stress indicating adaptive coping strategies. Stress-recovery conditions and symptom severity showed significant interactions. Facebook use was not effective for individuals with high symptom severity in contrast to non-digital strategies or for individuals with low symptom severity. The usage of social networks may be an adaptive strategy for coping with stress for some people, it is maladaptive for individuals having a problematic usage.

## Introduction

When demands (seemingly) exceed subjective resources individuals can perceive stress that manifests in psychological, behavioral, and physiological responses^1–3^. Since this happens frequently in human life, individuals have developed strategies to cope with stressors. In this context, recent research examined the relationship between stress perception and the use of social networks such as Facebook, Instagram, and Twitter^4–8^. While the use of social networks can have soothing effects (e.g., increasing self-esteem, well-being, perceived social support, life satisfaction^9–12^), it can also cause or perpetuate stress (e.g., through experiences of loneliness, depressive mood, anxiety^11,13–15^).

Drawing on the concrete association between stress and the use of social networks, a comprehensive overview by Wolfers and Utz^2^ emphasizes that three different functions can be distinguished: social networks (a) as stressor causing stress, (b) as resource which buffers stress, and (c) as coping strategy. Illustrating social-networks use as coping strategy, the transactional model of stress by Lazarus and Folkman^3^ could be used. It outlines that individuals choose different strategies to cope with situational demands, when they are evaluated as relevant and stressful. Wolfers and Schneider^16^ complement this theoretical approach and discuss that social-networks use represents such a coping strategy. It could result in beneficial or detrimental outcomes depending on the fit between strategy and situation^2^.

A coping-hypothesis (i.e., the use of social networks to overcome stress experiences) is also a prominent approach of recent research arguing that the excessive use of social networks might show addictive characteristics. Yet not being recognized as clinical disorder, researchers discuss the problematic use of social networks as potential “other specified disorder due to addictive behaviors”, which could be categorized in the Sect. 6C5Y in the International Classification of Diseases 11th (ICD-11) of the World Health Organization^17^. Accordingly, addiction-like use of social networks could be defined as being preoccupied with social networks, having a strong usage motivation, spending excessive amounts of time online, which overall lead to impairments in daily life and psychological well-being^18,19^.

Recent models that explain the development and maintenance of addictive behaviors therefore subsume addictive social-networks use as a specific subtype of addictive behaviors^20,21^.

Focusing on stress and addictive behaviors, the I-PACE model by Brand, et al.^22^, and its update^20^, illustrate stress vulnerability as a predisposing variable. The model also outlines how subjectively experienced stress and dysfunctional coping strategies may affect decision making and perception of situational cues. The interaction of these components could result in specific affective responses and diminished control over the use, which may then relate to negative consequences in daily life due to the use of specific applications. The authors highlight that the perception of internal and external triggers such as stress may influence affective and cognitive responses, for example urge for mood regulation, cue reactivity, craving, and attentional bias^20,22^. The cyclic passage of these processes favors the formation of habitual affective, cognitive, and behavioral responses to stressors which could also take the form of social-networks use itself to cope with stress^16^.

Consequently, theories around the use of social networks posit a stress-buffering-hypothesis (i.e., social-networks use as an adaptive strategy) as opposed to a coping-hypothesis (i.e., social-networks use as a maladaptive coping strategy favoring addictive behavior patterns in the long run). Both theoretical considerations find empirical support. Exemplarily, Rus and Tiemensma^23^ showed that if participants used Facebook or read magazines after stress induction, the subjectively perceived stress was significantly reduced. The physiological stress perception remained higher when using Facebook compared to reading magazines^23^. A study by Johnshoy, et al.^24^ also showed that the stress perception was similar between participants who used social networks and those who read magazines after stress induction. The authors concluded that Facebook use was beneficial for seeking social support online and relaxation after acute stress. Focusing on problematic behavior, Moretta and Buodo^25^ demonstrated that using Facebook for mood regulation impacts the general preference for online social interactions. This preference could lead to negative outcomes such as problematic use of Facebook. Further studies also emphasize that problematic social-networks use is related to increased stress experiences^4–6^.

However, less is known if it is still an adaptive strategy to cope with stress compared to other non-digital strategies when individuals experience negative consequences due to the use of social networks. Based on the hypothesized model by Wolfers and Utz^2^, the authors emphasize the importance of the coping-situation-fit, which could result in detrimental coping outcomes highlighting the differentiation of social-networks use as adaptive or maladaptive coping strategy. We conducted the current study with the aim of examining different strategies compared to social-networks use in stress recovery. Therefore, we used an experimental between-within-design with four groups: After stress induction, participants (1) used Facebook (experimental group 1; EG1), (2) read non-digital magazines (experimental group 2; EG2), or (3) waited without any distraction (control group 2; CG2). Another group, (4) simply waited without previous stress induction (control group 1; CG1). In line with Rus and Tiemensma^23^, we hypothesized that subjectively perceived stress is lower after using Facebook or reading magazines compared to before, i.e., immediately after stress induction, as well as compared to no form of distraction, an experimental condition which has been missing so far. Furthermore, based on the I-PACE model, we assumed that the form of stress recovery is affected by the symptom severity of problematic social-networks use. We hypothesized that the confrontation with a problem-related distractor such as Facebook use versus a non-problem-related distractor such as reading magazines leads to lower stress recovery in individuals with tendency towards a problematic social-networks use. In addition, we also investigated the effect of the problem-related distractor compared to no distractor (e.g., simply waiting), symptom severity, and its interaction on stress perception.

## Results

### Experimental manipulation

The mean values and the standard deviations for all four experimental conditions are shown in Table 1. We tested whether participants in the experimental conditions differed from each other in terms of perceived stress at time point 0 (t0; baseline) and at time point 2 (t2; after recovery) by using a mixed ANOVA with Greenhouse–Geisser correction. The results indicate no significant between-subject effect of the experimental conditions (*F*(100,3) = 1.52, *p* = 0.212, η_p_^2^ = 0.044). The within-subject factor (*F*(100,3) = 5.06, *p* = 0.027, η_p_^2^ = 0.048) and the interaction effect between subjectively perceived stress at t0 and t2 and the experimental conditions (*F*(100,3) = 3.25, *p* = 0.025, η_p_^2^ = 0.089) were significant. The post-hoc analysis of the interaction effect showed that only EG2 and CG1 significantly differed from each other at t2 (*p* = 0.034).

**Table 1:** Descriptive values of subjectively perceived stress over the course of the experiment for the overall sample and the different experimental conditions.

	Overall (N = 104)	Experimental group 1 (*n* = 25)	Experimental group 2 (*n* = 27)	Control group 1 (*n* = 26)	Control group 2 (*n* = 26)
STAI time point 0 (baseline)	37.43 (8.04)	38.72 (9.57)	36.19 (5.90)	37.50 (8.01)	37.42 (8.64)
STAI time point 1 (after induction)	50.80 (10.77)	51.56 (12.20)	47.85 (9.05)	53.12 (10.68)	–
STAI time point 2 (after recovery)	39.12 (9.27)	39.72 (10.56)	36.19 (6.74)	43.19 (10.25)	37.50 (0.09)

In addition, we investigated how subjectively perceived stress evolved over the course of the experiment, considering only the three conditions that included stress induction (EG1, EG2, CG1). An ANOVA with repeated measures indicated a significant within-subject effect in subjectively perceived stress (*F*(150,2) = 101.52, *p* < 0.001, η_p_^2^ = 0.575). The post-hoc analysis of the overall within-effect showed a significant increase from t0 to time point 1 (t1; after induction) indicating a successful stress induction (*p* < 0.001), a significant decrease from t1 to t2 emphasizing a successful stress reduction after the recovery period (*p* < 0.001) as well as a significant increase from t0 to t2 (*p* = 0.045). There were no significant between-subject effects (*F*(75,2) = 2.36, *p* = 0.102, η_p_^2^ = 0.059) nor a significant interaction effect between the stress perception and the experimental conditions (*F*(150,4) = 1.60, *p* = 0.179, η_p_^2^ = 0.041).

### Moderated regression analyses

**Figure 1:**
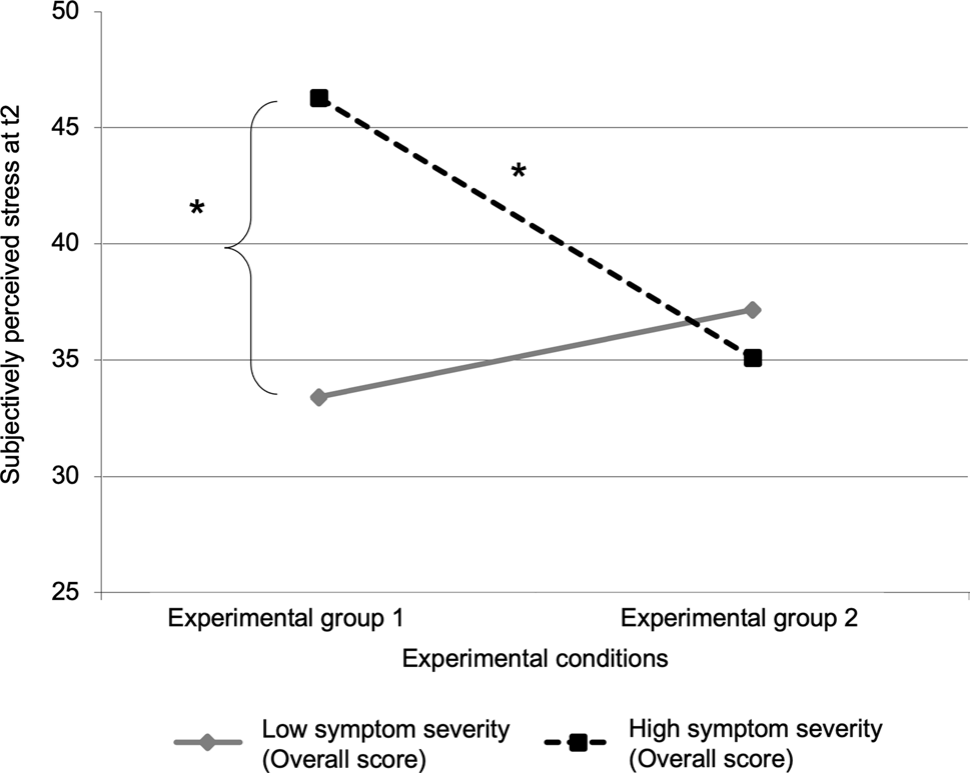
Interaction effects. The figure shows the simple slope analysis of the moderated regression analysis using the experimental conditions (Experimental group 1 and Experimental group 2), symptom severity, and their interactions as predictors and stress perception at t2 as dependent variable. *Note:* *p < 0.01.

To investigate the second hypothesis that using Facebook compared to reading magazines leads to lower stress recovery in individuals with tendency towards a problematic social-networks use, we used a moderated regression analysis for analyzing interaction effects between the experimental conditions and the symptom severity on perceived stress. The experimental conditions (EG1 and EG2), symptom severity, and the interaction term of both were used as predictors. Stress perception at t2 was used as dependent variable. The experimental conditions (*R*^2^ = 0.040, *F* = 2.10, *p* = 0.153) and symptom severity (Δ*R*^2^ = 0.064, Δ*F* = 3.49, *p* = 0.068) did not explain a significant amount of variance, but the interaction did (Δ*R*^2^ = 0.169, Δ*F* = 11.18, *p* = 0.002). In total, 27.3% of the variance could be explained (*R*^2^ = 0.273, *F(51,3)* = 6.02, *p* = 0.001). The simple slope analysis emphasized that participants with higher symptom severity showed higher stress perception when using Facebook as recovery compared to participants with higher symptom severity but reading magazines (*t* = 3.58, *p* = 0.001) as well as compared to participants with lower symptom severity recovering via Facebook (*t* = 3.87,* p* < 0.001) (see Fig. 1, see Table 2).

**Table 2:** Statistics of the coefficients of the moderated regression analyses predicting subjectively perceived stress at time point 2 by using two different experimental conditions each.

	*B*	*SE(B)*	*T*	*β*	*p*
*Model 1*					
Predictors					
Group (Experimental group 1 & Experimental group 2)	− 3.71	2.17	− 1.71	− 0.211	0.093
Symptom severity (Overall score)	0.45	0.18	2.46	0.305	0.018
2-way interaction					
Group x sIAT Overall score	− 1.23	0.37	− 3.34	− 0.415	0.002
*Model 2*					
Predictors					
Group (Experimental group 1 & Experimental group 2)	1.06	0.89	1.19	0.153	0.239
Symptom severity (Overall score)	0.36	0.21	1.66	0.220	0.104
2-way interaction					
Group x symptom severity	− 0.48	0.14	− 3.34	− 0.441	0.002

**Figure 2:**
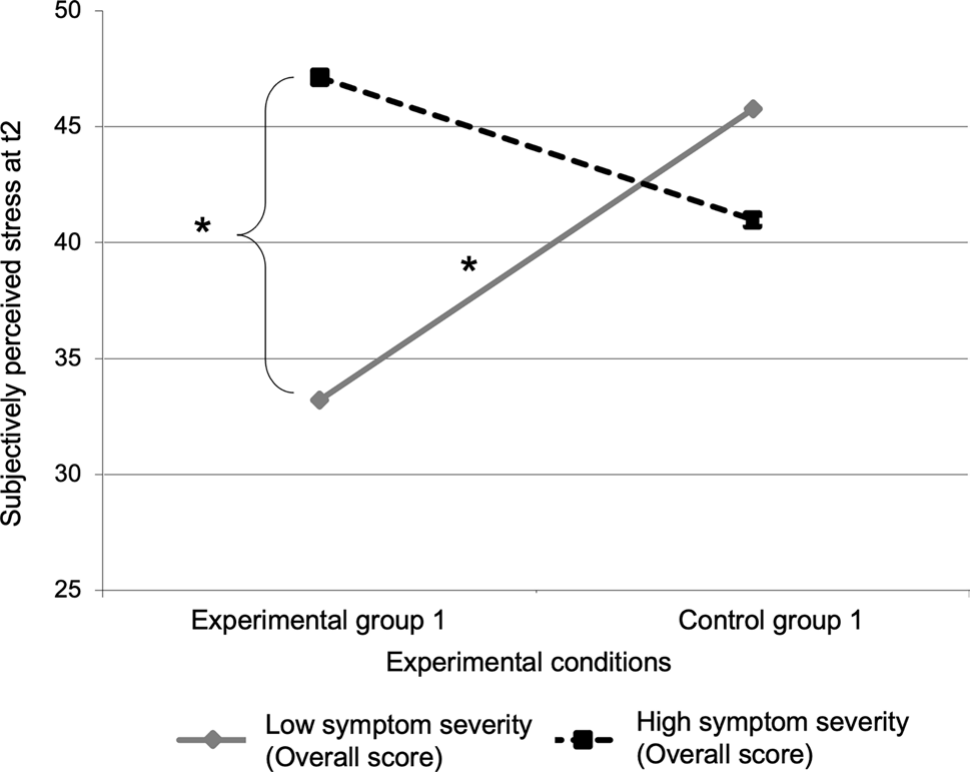
Interaction effects. The figure shows the simple slope analysis of the moderated regression analysis using the experimental conditions (Experimental group 1 and Control group 1), symptom severity, and their interactions as predictors and stress perception at t2 as dependent variable. *p < 0.01.

As additional analysis to further specify interaction effects, we investigated if the effect of using Facebook compared to waiting as form of recovery without any distractor on stress perception was affected by the tendency towards a problematic social-networks use as well. The experimental conditions (EG1 and CG1), symptom severity, and the interaction term were used, stress perception at t2 was the dependent variables. The experimental conditions (*R*^2^ = 0.028, *F* = 1.42, *p* = 0.239) and symptom severity (Δ*R*^2^ = 0.013, Δ*F* = 0.66, *p* = 0.422) had no significant effect, but the interaction significantly explained the variance of the changes in stress perception (Δ*R*^2^ = 0.184, Δ*F* = 11.13, *p* = 0.002). In total, 22.5% of the overall variance could be explained significantly (*R*^2^ = 0.225, *F(50,3)* = 4.54, *p* = 0.007). The simple slope analysis indicated that participants with higher symptom severity showed no higher stress perception when using Facebook compared to simply waiting (*t* = 1.30, *p* = 0.125). Only participants with lower symptom severity showed a better stress recovery when using Facebook compared to simply waiting (*t* = 3.24, *p* = 0.002). Again, participants with higher symptom severity showed higher stress perception compared to participants with lower symptom severity when using Facebook for recovery (*t* = 3.18, *p* = 0.003) (see Fig. 2, see Table 2).

As additional analyses we controlled the relationship between symptom severity and stress perception at the three measurement points for the three groups with stress induction (EG1, EG2, CG1). The bivariate correlation only showed a significant relationship between symptom severity and stress perception at t0 (*r* = 0.305, *p* = 0.007). Further relationships were not significant (*p*’s ≥ 0.076).

## Discussion

The aim of the current study was to investigate if social-networks use could be highlighted as adaptive or maladaptive coping strategy compared to non-digital coping strategies. We also hypothesized that stress recovery by using social networks was affected by the symptom severity of a problematic social-networks use. The results showed that using Facebook or reading magazines leads to significant reduction of previously induced stress in contrast to conditions without any form of distraction. Investigating the interaction of different stress-recovery strategies and symptom severity, in individuals suffering problematic use of social networks Facebook use did not result in lower stress perception compared to individuals with low symptom severity or non-digital strategies. The use of social networks can thus be declared as both an adaptive and maladaptive strategy, whereby it seems important to pay more attention to possible negative consequences due to problematic use.

The results addressing stress recovery and Facebook use are, overall, in line with Rus and Tiemensma^23^ who illustrated psychological recovery of participants by using Facebook or reading magazines in a comparable setting. Going beyond this, our results outline that both conditions showed higher effects on stress recovery compared to no form of distraction. Some type of distraction might be helpful at least on a subjective level. However, it might be worth to control the potential of recovery without any distraction since the results on a physiological level could differ^23^. This seems to be relevant especially when stress has been experienced. Letting time pass or simply waiting was not as effective as being confronted with any form of distraction. Wolfers and Utz^2^ highlighted that the use of social networks could be an adaptive strategy when dealing with acute stress. It is based on the adaption of the transactional model of stress and coping^3^ outlining that stress could trigger social-networks use as coping strategy, which could be effective if strategy and situation fit^2,16^. The current results demonstrate that the effectiveness of using social networks as coping strategy depends on the level of problematic social-networks use. The use of the problem-related application such as Facebook affected the stress recovery leading to still higher stress perception in individuals with higher symptom severity compared to (a) individuals with lower symptom severity and (b) to other forms of recovery such as reading magazines or simply waiting. Interestingly, participants with lower symptom severity showed better recovery when using Facebook compared to recovery without any distraction. Overall, the usage of social networks may be an adaptive strategy to cope with stress for individuals having no problems with regulating their social-networks use. For individuals with problematic usage, the use of social networks in situations of stress experiences seems to be maladaptive. Therefore, it may be worth considering that previous study results and theoretical assumptions should be reviewed critically since interindividual differences, personal characteristics, or media-use behaviors seem to have effects on whether media use is an adaptive or maladaptive coping strategy.

The result that social-networks use can be a maladaptive stress-coping strategy for individuals with problematic usage is an important finding. It shows that stress experiences are considered a driving factor for problematic usage. This means that there could be a self-reinforcing effect in individuals with a tendency towards problematic social-networks use: Stress may foster the usage of social networks, but other than expected; the actual use may not decrease the level of stress, which may keep users using social networks even longer. This fits the theoretically argued vicious circle of the addiction process, for example in the I-PACE model^20,22^, arguing that dysfunctional coping mechanisms could enhance the risk of an addiction-like behaviors. Beyond this, the I-PACE model additionally emphasizes that the experience of stress could reinforces the use of a specific online application. Individuals might experience a form of gratification as positive reinforcement or compensation for negative emotions. In the long term, this could implicate alternative strategies for dealing with stress or negative emotions are no longer used, whereas the use of specific applications is increasingly linked to the expectation of avoiding negative emotions or experience positive feelings. It could be assumed that, unlike for non-problematic use, the specific application is no longer an adaptive coping strategy since negative consequences in daily life have already been experienced due to the use of the application itself. This can be particularly relevant if other mechanisms and processes, as suggested in the I-PACE model, are involved. Affective and cognitive components such as attentional bias, cue reactivity, craving, and reduced inhibitory control play an important role in the interaction between stress, coping with stress, and the use of social networks^20,22^. The experience of stress and the (unconscious) perception of application-specific triggers could lead to higher craving or the desire to use social networks. Cue reactivity and craving are already shown as risk factors of a problematic social-networks use^26,27^. The interaction could explain why individuals with higher symptom severity do not experience the use itself as stress-reducing or recovering. However, the interaction of the different components and mechanisms needs to be investigated in future studies^28^.

The interplay of the different components in the addiction process has also implications for prevention and related maladaptive coping strategies. Keeping the reinforcement mechanisms proposed in the I-PACE model in mind, it seems to be important to address potential mechanisms when dealing with stress which could result in an addictive behavior. Maladaptive coping strategies and dysfunctional emotion regulation as well as using social networks for mood management have been shown to be risk factors of a problematic social-networks use^19,25,29^. Functional emotion regulation and conflict as well as mood management, which is not exclusively online, but where alternative strategies are sought, seem to represent a preventive mechanism. However, in some situations social networks could be the targeted strategy for finding social support and belonging needed^16,30^. In such cases, it seems to be important to distinguish when the use itself is functional and fits the situational requirements leading to beneficial outcome and when it is not^2^ or even affected by specific individual characteristics. For a better understanding, a more detailed investigation of the usage behaviors and relevant interindividual differences when dealing with stress is needed.

However, some limitations should be considered. First of all, it seems to be worth to discuss Facebook and its usage:  In the current study, objective usage data of social networks have not been assessed, which could provide further insights into the general usage behavior. Moreover, participants were not allowed to choose their preferred social network. For some users, Facebook may not have been the social network they would normally use when experiencing negative emotions or stress. This could therefore be one reason why some of the reactions shown were correspondingly low. Nevertheless, similar mechanisms might be relevant to other social networks as well. In addition, the content of Facebook could have affected the stress recovery as well, for example when the participants are confronted with negative or even positive information or messages. Future studies should address the content when using social networks to include possible confounding effects^2^. As second limitation, we have to discuss further methodological issues: it should be mentioned that we have not included physiological measurements. Since Rus and Tiemensma^23^ showed differences between psychological and physiological measurements, the systematic investigation of physiological reactions using EEG or cortisol in individuals with problematic social-networks use is highly needed. Since our measures were subjective, future studies should add physiological measurements to overcome this limitation. This could also be a validation if the participants really perceived the situation as stressful or anxious or if it was just a bias due to the subjective questioning. Regarding the choice of questionnaires, the STAI has originally been used to assess state anxiety, and even if its association with acute stress perception was emphasized^1,31^, further validation of stress perception and including a baseline assessment, for example the trait anxiety scale as control variable, is needed in future studies. We also would like to stress out that the assessment of problematic social-networks use is still challenging since no official diagnosis or measurement standard exist, which could lead to an overestimation of problematic behavior. In the following study, we attempted to standardize, so only one specific network was manipulated as a condition, while symptom severity was recorded in general. Nevertheless, research faces the challenge of finding a balance in this context between querying the specific social network relevant to the individuals and recording usage in general^32^.

### Conclusion

The current study supports previous findings showing that the use of social networks, like Facebook, and reading non-digital magazines can decrease subjective stress after stress induction. This may indicate that both are adaptive coping strategies when dealing with potential subjectively perceived stress. Interindividual differences, however, are important to consider when making conclusions about the effectiveness of using social networks as coping strategy. Using social networks for stress reduction can be maladaptive in individuals with addiction tendency. Nevertheless, it should be considered that mainly subjective measurements were taken, which may not be representative for the biopsychological state of stress. In future, it would be helpful to complement the study with psychophysiological markers (e.g., electrodermal activity, salivary cortisol) and to conduct the study in a clinical sample with individuals suffering from negative consequences due to the use of social networks. Both would give more detailed insights into the mechanisms identified in the current study. Future studies should also investigate the interactions of maladaptive coping strategies, interindividual differences, and affective and cognitive processes such as attentional bias, implicit associations, cue reactivity, and craving when dealing with stress as potential reinforcement mechanisms and their role in developing a problematic use of social networks.

## Methods

### Participants and study design

In this study, 104 participants (85 females, 19 males) aged between 18 and 45 years (*M* = 22.06, *SD* = 5.67) have been included who mentioned to use social networks regularly. Seventy-five (72.15%) participants reported a non-problematic, 20 (19.24%) participants reported a problematic, and nine participants (8.66%) reported a pathological use of social networks (see Measurement section).

The participants were recruited at the University of Duisburg-Essen, Germany. The study was conducted in a laboratory, individual setting. It has been approved by the ethics committee of the Department for Computer Science and Applied Cognitive Science at the Faculty of Engineering of the University of Duisburg-Essen, Germany. All the methods were performed in accordance with the Declaration of Helsinki. All participants gave written informed consent by signing a declaration of consent after they have been informed about the procedure. After the end of the study, the participants received further information about the study content and had the opportunity to ask questions. Students received credits points for participating.

The study followed a between-within design with four experimental conditions (between-factor) and three measurement time points (within-factor). After instructions of the setting, the participants were asked to fill in the questionnaire assessing tendency towards a problematic use of social networks, which has been analyzed immediately. This score was used along with age and gender to pseudo-randomly assign the participants to one of the four groups to control for potential confounding variables (see Table 3): Experimental group 1 (EG1; stress reduction via Facebook), experimental group 2 (EG2; stress reduction via magazines), control group 1 (CG1; stress reduction via waiting), control group 2 (CG2; only waiting without stress induction). In the first experimental condition (EG1), participants were asked to log in and open their Facebook profile. They were allowed to read through their news feeds and posts which have been uploaded, but not to response. The second experimental condition (EG2) was defined as non-digital recovery: Participants were asked to choose between different magazines (e.g., boulevard, fashion, finance, cars) and to read through them. In the control conditions (CG1 & CG2), the recovery phase consisted of waiting and the participants should sit quietly in the chair without using a digital device or other forms of distraction. Each recovery phase lasted exactly five minutes. The study took place in a university laboratory room where participants sat quietly on a desk chair and at a desk with a computer screen. In addition to daylight, the room was brightly illuminated with electronic light. Ambient noise from the adjacent floor such as soft voices or footsteps could not be excluded. The participants were not allowed to interact with others.

**Table 3:** Overview of the sample and group descriptions regarding age, gender, and tendency towards a problematic social-networks use including group comparisons.

	Overall (N = 104)	Experimental group 1 (*n* = 25)	Experimental group 2 (*n* = 27)	Control group 1 (*n* = 26)	Control group 2 (*n* = 26)	Group comparisons
Age	22.06 (5.67)	21.84 (4.45)	22.11 (5.99)	22.58 (6.24)	21.69 (6.06)	*F*(100,3) = 0.12, *p* = 0.949, η_p_^2^ = 0.004
Gender (f/m/d)	85/19/0	21/4/0	21/6/0	20/6/0	23/3/0	Chi^2^(3) = 1.56, *p* = 0.668
Symptom severity (Overall score)	26.09 (6.57)	25.84 (5.67)	26.19 (6.43)	26.62 (7.22)	25.69 (7.17)	*F*(100,3) = 0.10, *p* = 0.961, η_p_^2^ = 0.003
Symptom severity (Factor 1)	16.18 (4.45)	15.64 (3.94)	16.56 (3.98)	16.73 (5.02)	15.77 (4.89)	*F*(100,3) = 0.39, *p* = 0.763, η_p_^2^ = 0.011
Symptom severity Factor 2	9.90 (2.91)	10.20 (2.80)	9.63 (3.08)	9.88 (2.63)	9.92 (3.24)	*F*(100,3) = 0.16 *p* = 0.921, η_p_^2^ = 0.005

**Figure 3:**
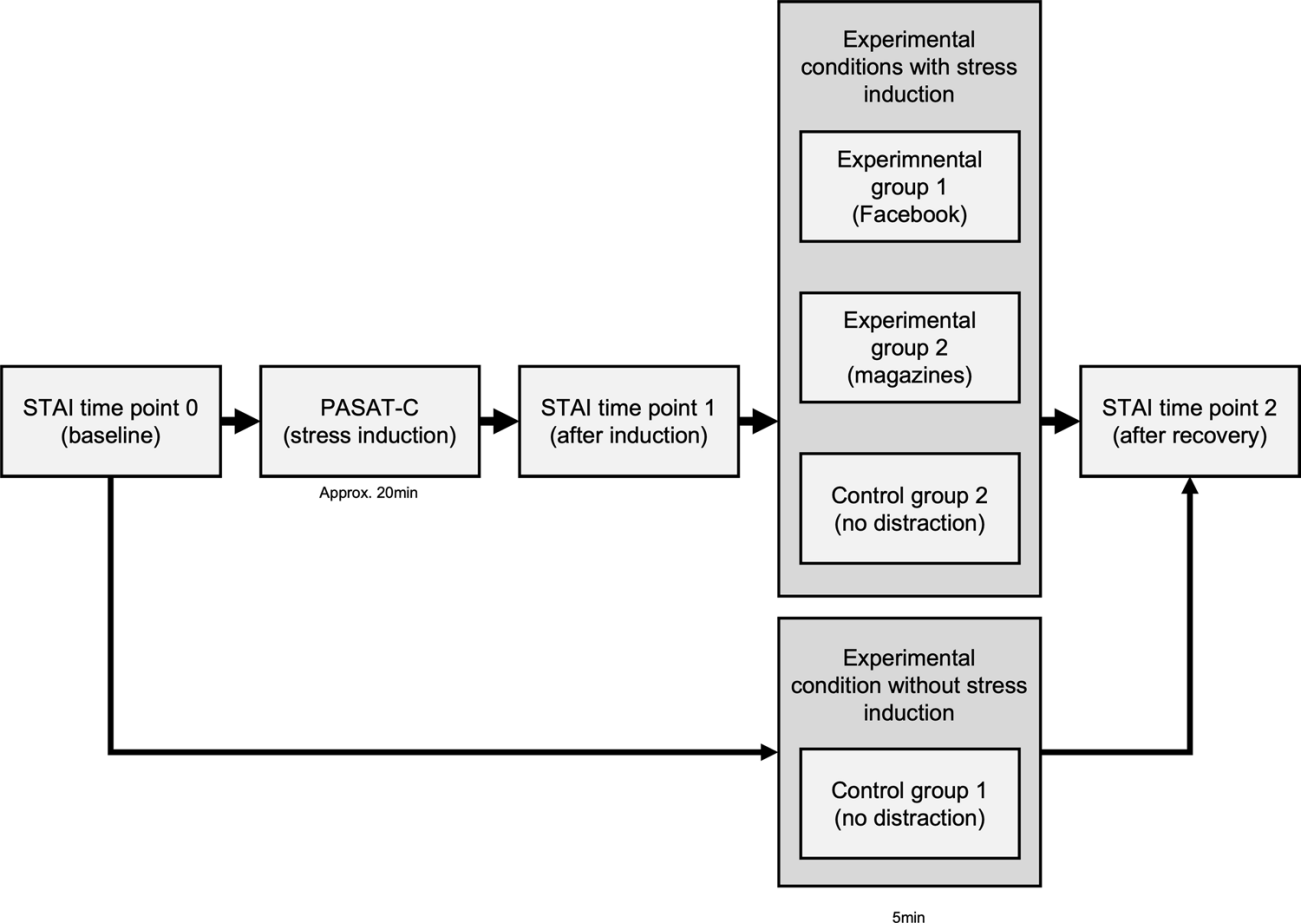
Study design. This figure shows the procedure of the study for the four different conditions. Participants of condition CG2 end up in the recovery phase directly after measurement of subjectively perceived stress (STAI time point 0), while subjects of condition EG1, EG2, CG1 must process the PASAT-C as stress induction after measurement of subjectively perceived stress (time point 0), stress perception (STAI time point 1) was assessed one more time and then are assigned to one of the three different conditions. For all participants there was an additional measurement of subjectively perceived stress (STAI time point 2) after the recovery condition. *Note:* STAI = State and Trait Anxiety Inventory, PASAT-C = Paced Auditory Serial Addition Task. After time point 0 the execution of the PASAT-C took 20 min and then the measurement of time point 1 has been done, followed by the experimental conditions (5 min). After that, the measurement of time point 2 was carried out. Only in CG1 the survey lasted shorter, so that between t0 and t2 was only 5 min.

At t0, subjectively perceived stress was measured in all subjects using the State and Trait Anxiety Inventory. Participants in EG1, EG2, and CG1 subsequently underwent the stress induction followed by the second measurement of subjectively perceived stress (t1). They then recovered in one of three different conditions. Participants assigned to CG2 did not process the stress induction and began with the recovery phase directly after t0. After the recovery phase, the last measurement of subjectively perceived stress was done (t2) (see Fig. 3).

### Instruments

#### Tendency towards problematic social-networks use

We used the short Internet Addiction Test modified for social networks to investigate subjectively perceived symptoms of problematic use of social networks^33^. The questionnaire consists of twelve items, which must be rated on a five-point Likert scale (1 = never to 5 = very often). It includes two factors each containing of six items: loss of control/time management and social problems/craving. An overall sum score was calculated ranging from twelve to 60, where higher scores indicate a higher tendency towards problematic social-networks use. Participants with sum scores > 30 were identified as users with problematic use and participants with sum scores > 37 as users with pathological use^34^. The internal consistency of the sum score was good (Cronbach’s Alpha = 0.849). This is based on the subjective self-report of the participants, as no recognized diagnostic criteria are yet available, and an objective assessment could not be made.

#### Subjectively perceived stress

The State and Trait Anxiety Inventory (STAI) by Spielberger^35^ normally assess trait and state anxiety. We used the STAI-State scale assessing the current emotional state, since it has been used to measure subjectively perceived stress^1,36^. In addition, the study by Julian^31^ outlines that it is a valid measure to assess a current negative state (e.g., anxiety, negative stress), within STAI state shows stronger validity to experienced stress than to currently experienced anxiety in comparative studies^31^. The scale consists of 20 items, which must be rated on four-point Likert scale (1 = not at all to 4 = very much). A sum score was calculated, where a higher score represents higher subjectively perceived stress. The questionnaire was used third times: as baseline (STAI t0), after stress induction (STAI t1), and after the experimental conditions (STAI t2). The internal consistencies of the scores were good (Cronbach’s Alpha at t0 = 0.885; Cronbach’s Alpha at t1 = 0.919; Cronbach’s Alpha at t2 = 0.919).

#### Stress induction

The Paced Auditory Serial Addition Task PASAT-C^37^ has been used to induce stress. Participants were asked to add numbers as fast as possible that were serially presented on a screen. An aversive sound is played when adding an incorrect number or being too late. During the task, the difficulty (short inter-stimulus interval) and level length (from three to ten minutes) increase. Overall, it took 20 min. The task has successfully been used to induce stress^1,38,39^.

### Statistical analysis

Statistical analyses were carried out with SPSS 27.0 (IBM statistics for Mac). Group differences in gender were analyzed using the Chi^2^-Test. Addressing the hypotheses, we used analyses of variance (ANOVA) applied on subjective stress measure with the time points as within-subject factor and the experimental conditions as between-subject factor. For multiple comparisons during post-hoc analysis, Bonferroni correction was used (Section "Experimental manipulation"). Bivariate relationships between the variables were analyzed by Pearson’s correlations. Investigating the effect of experimental conditions (EG1, EG2) and symptom severity on stress perception at t2, we used multiple hierarchical regression analyses, which included interaction effects with all predictor variables being mean-centered and by calculating the interaction term of the predictor variables^40,41^. Simple slope analyses were done analyzing significant interaction effects whereby estimated mean scores based on the regression’s coefficients were used. Subjects were grouped one standard deviation below and above the group’s means: Low values represented estimates for subjects with values one standard deviation below the group mean; high values represented estimates for subjects with values one standard deviation above the group mean. To be able to evaluate the significance of the effects retrospectively, we calculated a sensitivity power analysis using ANOVA with repeated measures (within-between-interaction) by using G*Power 3.1. Considering the total sample size (N = 104), four experimental conditions, and three measurement points as well as the standard parameter (Alpha-error = 05; Power = 0.95), the results illustrate a critical *F* = 2.14 and the effect size *f* = 0.15 indicating a small to medium effect.

## Data Availability

The datasets used and/or analyzed during the current study available from the corresponding author on reasonable request.

## References

[1] Starcke K, Wiesen C, Trotzke P, Brand M (2016). Effects of acute laboratory stress on executive functions. Front. Psychol..

[2] Wolfers LN, Utz S (2022). Social media use, stress, and coping. Curr. Opin. Psych..

[3] Lazarus RS, Folkman S (1984). Stress, Appraisal, and Coping.

[4] Hou X (2017). Psychological resilience can help combat the effect of stress on problematic social networking site usage. Pers. Individ. Dif..

[5] Brailovskaia J, Rohmann E, Bierhoff HW, Schillack H, Margraf J (2019). The relationship between daily stress, social support and Facebook addiction disorder. Psychiat. Res..

[6] Brailovskaia J, Truskauskaite-Kuneviciene I, Kazlauskas E, Margraf J (2023). The patterns of problematic social media use (SMU) and their relationship with online flow, life satisfaction, depression, anxiety and stress symptoms in Lithuania and in Germany. Curr. Psychol..

[7] Ryan T, Chester A, Reece J, Xenos S (2014). The uses and abuses of Facebook: A review of Facebook addiction. J. Behav. Addict..

[8] Boyd DM, Ellison NB (2007). Social network sites: Definition, history, and scholarship. J. Comput.-Mediat. Commun..

[9] O’Reilly M (2018). Is social media bad for mental health and wellbeing? Exploring the perspectives of adolescents. Clin. Child Psychol. Psychiat..

[10] Lee G, Lee J, Kwon S (2011). Use of social networking sites and subjective well-being: A study in South Korea. Cyberpsychol. Behav. Soc. Netw..

[11] Valkenburg PM (2022). Social media use and well-being: What we know and what we need to know. Curr. Opin. Psych..

[12] Liu D, Baumeister RF, Yang CC, Hu B (2019). Digital communication media use and psychological well-being: A meta-analysis. J. Comput.-Mediat. Commun..

[13] Cunningham S, Hudson CC, Harkness K (2021). Social media and depression symptoms: A meta-analysis. Res. Child Adolesc. Psychopathol..

[14] Huang C (2020). A meta-analysis of the problematic social media use and mental health. Int. J. Soc. Psychiat..

[15] Hussain Z, Wegmann E, Yang H, Montag C (2020). Social networks use disorder and associations with depression and anxiety symptoms: A systematic review of recent research in China. Front. Psychol..

[16] Wolfers LN, Schneider FM (2020). Using media for coping: A scoping review. Commun. Res..

[17] Brand M (2022). Which conditions should be considered as disorders in the international classification of diseases (ICD-11) designation of „other specified disorders due to addictive behaviors“?. J. Behav. Addict..

[18] Andreassen CS, Pallesen S (2014). Social network site addiction: An overview. Curr. Pharm. Des..

[19] Hussain Z, Wegmann E, Griffiths MD (2021). The association between problematic social networking site use, dark triad traits, and emotion dysregulation. BMC Psychol.

[20] Brand M (2019). The interaction of person-affect-cognition-execution (I-PACE) model for addictive behaviors: Update, generalization to addictive behaviors beyond Internet-use disorders, and specification of the process character of addictive behaviors. Neurosci. Biobehav. Rev..

[21] Wegmann E, Brand M (2019). A narrative overview about psychosocial characteristics as risk factors of a problematic social networks use. Curr. Addict. Rep..

[22] Brand M, Young KS, Laier C, Wölfling K, Potenza MN (2016). Integrating psychological and neurobiological considerations regarding the development and maintenance of specific Internet-use disorders: An interaction of person-affect-cognition-execution (I-PACE) model. Neurosci. Biobehav. Rev..

[23] Rus HM, Tiemensma J (2017). Social media under the skin: Facebook use after acute stress impairs cortisol recovery. Front. Psychol..

[24] Johnshoy Q (2020). Social media use following exposure to an acute stressor facilitates recovery from the stress response. Physiol. Behav..

[25] Moretta T, Buodo G (2018). Modeling problematic Facebook use: Highlighting the role of mood regulation and preference for online social interaction. Addict. Behav..

[26] Wegmann E, Stodt B, Brand M (2018). Cue-induced craving in Internet-communication disorder using visual and auditory cues in a cue-reactivity paradigm. Addict. Res. Theory.

[27] Leng Y (2019). The craving and excitement of social networking sites addicts: Based on cue-reactivity. Front. Psychol..

[28] Brand M (2021). Addiction research unit: Affective and cognitive mechanisms of specific Internet-use disorders. Addict. Biol..

[29] Elhai JD, Levine JC, Hall BJ (2019). The relationship between anxiety symptom severity and problematic smartphone use: A review of the literature and conceptual frameworks. J. Anxiety Disord..

[30] Frison E, Eggermont S (2015). The impact of daily stress on adolescents’ depressed mood: The role of social support seeking through Facebook. Comput. Human Behav..

[31] Julian LJ (2011). Measures of anxiety: State-trait anxiety inventory (STAI), Beck Anxiety Inventory (BAI), and Hospital Anxiety and Depression Scale-Anxiety (HADS-A). Arthritis Care Res. Hoboken.

[32] Cataldo I, Billieux J, Esposito G, Corazza O (2022). Assessing problematic use of social media: Where do we stand and what can be improved?. Curr. Opin. Behav. Sci..

[33] Wegmann E, Stodt B, Brand M (2015). Addictive use of social networking sites can be explained by the interaction of Internet use expectancies, internet literacy, and psychopathological symptoms. J. Behav. Addict..

[34] Pawlikowski M, Altstötter-Gleich C, Brand M (2013). Validation and psychometric properties of a short version of Young's Internet Addiction Test. Comput. Human Behav..

[35] Spielberger CD (1972). State-Trait Anxiety Inventory. Prof. Psychol..

[36] Greene J, Cohen D, Siskowski C, Toyinbo P (2017). The relationship bewtee family caregiving and the mental health of emerging young adult caregivers. J. Behav. Health Serv. Res..

[37] Lejuez, C. W., Kahler, C. W. & Brown, R. A. A modified computer version of the Paced Auditory Serial Addition Task (PASAT) as a laboratory-based stressor. *Behav. Ther.* (2003).

[38] Mathias CW, Stanford MS, Houston RJ (2004). The physiological experience of the Paced Auditory Serial Addition Task (PASAT): Does the PASAT induce autonomic arousal?. Arch. Clin. Neuropsychol..

[39] Reinhardt T, Schmahl C, Wüst S, Bohus M (2012). Salivary cortisol, heart rate, electrodermal activity and subjective stress responses to the Mannheim multicomponent stress test (MMST). Psychiat. Res..

[40] Aiken LS, West SG, Reno RR (1991). Multiple Regression: Testing and Interpreting Interactions.

[41] Cohen J, Cohen P, West SG, Aiken LS (2003). Applied Multiple Regression/correlation Analysis for the Behavioral Science.

